# Increased sample volume and use of quantitative reverse-transcription PCR can improve prediction of liver-to-blood inoculum size in controlled human malaria infection studies

**DOI:** 10.1186/s12936-015-0541-6

**Published:** 2015-01-28

**Authors:** Susanne H Hodgson, Alexander D Douglas, Nick J Edwards, Domtila Kimani, Sean C Elias, Ming Chang, Glenda Daza, Annette M Seilie, Charles Magiri, Alfred Muia, Elizabeth A Juma, Andrew O Cole, Thomas W Rampling, Nicholas A Anagnostou, Sarah C Gilbert, Stephen L Hoffman, Simon J Draper, Philip Bejon, Bernhards Ogutu, Kevin Marsh, Adrian VS Hill, Sean C Murphy

**Affiliations:** The Jenner Institute, University of Oxford, Oxford, UK; Department of Laboratory Medicine and Center for Emerging and Re-Emerging Infectious Diseases, University of Washington (UW), 750 Republican St., E633, Seattle, WA 98109 USA; Kenya Medical Research Institute - Wellcome Trust, Centre for Geographical Medical Research (Coast), Kilifi, Kenya; Centre for Clinical Research, Kenya Medical Research Institute, Nairobi, Kenya; Centre for Research in Therapeutic Sciences, Strathmore University, Nairobi, Kenya; Sanaria, Inc, Rockville, MD USA

**Keywords:** *Plasmodium falciparum*, 18S rRNA, PCR, RT-PCR, Standards, Controls, Calibrators, Pre-erythrocytic

## Abstract

**Background:**

Controlled human malaria infection (CHMI) studies increasingly rely on nucleic acid test (NAT) methods to detect and quantify parasites in the blood of infected participants. The lower limits of detection and quantification vary amongst the assays used throughout the world, which may affect the ability of mathematical models to accurately estimate the liver-to-blood inoculum (LBI) values that are used to judge the efficacy of pre-erythrocytic vaccine and drug candidates.

**Methods:**

Samples were collected around the time of onset of pre-patent parasitaemia from subjects who enrolled in two different CHMI clinical trials. Blood samples were tested for *Plasmodium falciparum* 18S rRNA and/or rDNA targets by different NAT methods and results were compared. Methods included an ultrasensitive, large volume modification of an established quantitative reverse transcription PCR (qRT-PCR) assay that achieves detection of as little as one parasite/mL of whole blood.

**Results:**

Large volume qRT-PCR at the University of Washington was the most sensitive test and generated quantifiable data more often than any other NAT methodology. Standard quantitative PCR (qPCR) performed at the University of Oxford and standard volume qRT-PCR performed at the University of Washington were less sensitive than the large volume qRT-PCR, especially at 6.5 days after CHMI. In these trials, the proportion of participants for whom LBI could be accurately quantified using parasite density value greater than or equal to the lower limit of quantification was increased. A greater improvement would be expected in trials in which numerous subjects receive a lower LBI or low dose challenge.

**Conclusions:**

Standard qPCR and qRT-PCR methods with analytical sensitivities of ~20 parasites/mL probably suffice for most CHMI purposes, but the newly developed large volume qRT-PCR may be able to answer specific questions when more analytical sensitivity is required.

## Background

The controlled human malaria infection (CHMI) model is a well-established system to determine the efficacy of drug and vaccine candidates early in their clinical development process. In this model, healthy human subjects are infected with *Plasmodium* spp. either through the bite of infectious mosquitoes or by needle-based administration of cryopreserved sporozoites or infected red blood cells [1-4]. Upon diagnosis of patent parasitaemia by thick blood smear (TBS), or in some cases pre-patent parasitaemia by nucleic acid tests (NATs), subjects are treated with approved curative therapy and followed to ensure parasite clearance and recovery.

Various NATs have been used to detect and quantify pre-microscopy patent blood-stage parasitaemia. The most common targets are the 18S rRNA-coding genes (rDNA) or the asexual-type 18S rRNAs themselves, which can be detected by nested polymerase chain reaction (PCR), quantitative PCR (qPCR), nucleic acid sequence-based amplification (NASBA) and quantitative reverse transcription PCR (qRT-PCR), as recently reviewed [5]. When applied in the post-CHMI setting, blood-stage infections can often be detected by NATs two to five days earlier than by TBS, with highly sensitive NAT assays becoming positive around Day 7 post-CHMI (D7.0) when standard CHMI doses of five infectious mosquito bites are used [6-10].

Modelling of NAT data allows calculation of the parasite multiplication rate (PMR) of blood-stage parasites as well as estimation of the number of parasites released from the liver into the blood (liver-to-blood inoculum (LBI)) [11]. LBI is a measure of the efficacy of drugs and vaccines designed to reduce or eliminate liver-stage development of the pre-erythrocytic malaria parasite. Parasite release from the liver after CHMI is thought to start on D6 but not be complete until D7.5 [12,13]; one report indicated detection as early as D5 post-CHMI [10]. Since many samples are NAT negative at these early timepoints, direct measurement of LBI is not possible so modelling methods are usually employed to estimate LBI [11]. If it were possible to enhance NAT sensitivity at these early timepoints to allow direct measurement of LBI, the accuracy and confidence of LBI measures would be markedly increased.

Each ring-stage parasite genome contains five 18S rDNA genes, and DNA-based NATs can therefore detect 1–5 of those copies depending on sequence specificity. At the published 5 parasites/mL (p/mL) lower limit of detection (LLD) for the Oxford qPCR method [9], 15 copies of the Oxford assay’s target sequence are present in 1 mL of whole blood (three copies per parasite genome). For this assay, the LLD is the lowest target density where rDNA from the 0.5 mL whole blood extraction is likely to be present in the 10% eluate volume transferred into each qPCR reaction, resulting in a just-detectable qPCR signal with a cycle threshold (C_T_) of ~41 cycles. Because of the low number of target sequences per parasite, this LLD is somewhat higher than the parasite density at which it becomes probable that at least one parasite will be present in the blood from which DNA is extracted. The lower limit of quantification (LLQ) is yet again higher than the LLD.

In contrast, in the University of Washington (UW) qRT-PCR method [14], ~3,500 target 18S rRNA copies are present per ring-stage parasite. At the 20 p/mL published LLD, the quanta of rRNA for a single parasite in 0.05 mL of whole blood leads to qRT-PCR detection at a C_T_ of ~33.5 cycles. Thus, in qRT-PCR, the sample volume is the primary limiting factor for the LLD since each parasite contains a readily-detectable number of targets. Since the rRNA is ~3 logs more abundant than the parent rDNA genes, qRT-PCR can be performed on small blood volumes and yet achieve sensitivities comparable to that of larger volume qPCR. With these issues in mind, assay modifications were tested in an attempt to increase the sensitivity of qPCR and qRT-PCR to directly measure LBI on D6 post-CHMI.

To increase the sensitivity of Oxford qPCR to detect infections at <5 p/mL, larger volumes of blood could be processed and/or a larger proportion of extracted DNA could be used per PCR reaction. In either case, enhanced sensitivity would require maintenance of a reasonable ratio of parasite DNA to blood-derived PCR inhibitors -- adequate removal of human DNA and other PCR inhibitors such as haemoglobin from large samples can be challenging (ADD, unpublished observations). Because of the low number of target copies per parasite, this problem cannot be overcome readily by diluting the extracted DNA. In addition, it is relatively inconvenient to collect, store and process 5–10 mL volumes of whole blood for PCR-based diagnostics.

To enhance the analytical sensitivity of qRT-PCR to detect infections at <20 p/mL, a ten-fold larger sample could be collected (0.5 mL *vs* 0.05 mL) provided that it was preserved in ten-fold more lysis buffer (20 mL *vs* 2 mL). With this approach, the LLD could theoretically reach 2 p/mL. If a 0.5 mL blood aliquot contained a single parasite with ~3,500 target copies, ~170 rRNA copies would be aliquoted into the qRT-PCR reaction as compared to only 2–5 rDNA copies. Here, this approach was tested using duplicate 0.5 mL qRT-PCR samples (hereafter referred to as ‘large volume’ qRT-PCR) alongside other approaches including the 0.5 mL Oxford qPCR method, the standard 0.05 mL qRT-PCR method and a qRT-PCR approach for 0.05 mL blood on dried blood spot (DBS) cards.

## Methods

### Clinical samples

Whole blood samples were obtained from subjects enrolled in two Institutional Review Board-approved CHMI trials: VAC052 and KCS.

VAC052 was a CHMI trial conducted at the Centre for Clinical Vaccinology & Tropical Medicine, Oxford, UK to assess the efficacy of candidate pre-erythrocytic viral vectors chimpanzee adenovirus 63 (ChAd63) and modified vaccinia virus Ankara (MVA) vectors containing multiple epitopes - thrombospondin related adhesion protein (ME-TRAP), circumsporozoite protein (CS) and apical membrane antigen 1 (AMA1) administered in combination (Clinicaltrials.gov NCT01739036) (NAA *et al*., in preparation). The study consisted of three groups: Group 1 immunized with ChAd63-MVA ME-TRAP + CSP (*n = 13*), Group 2 immunized with ChAd63-MVA ME-TRAP + CSP + AMA1 (*n = 13*) and Group 3 unimmunized infectivity controls (*n = 6*). Volunteers underwent CHMI with five *Plasmodium falciparum*-infected (3D7) mosquito bites. Sporozoite carriage was confirmed by dissection and microscopy following blood meals and each mosquito was required to display evidence of a blood meal and ≥11 sporozoites in its salivary glands. VAC052 was approved by the National Research Ethics Service Committee South Central – Oxford A, UK and the Western Institutional Review Board, USA. The study was conducted from January to October 2013.

The KCS study was a CHMI trial conducted at the Kenyan Medical Research Institute (KEMRI) Centre for Clinical Research, Nairobi, Kenya (Pan African Clinical Trial Registry PACTR20121100033272) [15]. Volunteers with varying degrees of prior exposure to malaria were infected using intramuscular administration of different doses of aseptic, purified, cryopreserved *P. falciparum* (NF54) sporozoites (PfSPZ Challenge, Sanaria, Inc.) [8,9,16]. The KCS study was approved by KEMRI Ethics Review Committee, Kenya and the Oxford Tropical Research Ethics Committee, UK and was conducted under a US Food and Drug Administration Investigational New Drug Application (FDA IND #14267). The study was conducted from March to August 2013.

In both studies, CHMI occurred on D0. For VAC052, Oxford qPCR assay and TBS microscopy were performed at D6.5, D7.0, D7.3, and D7.5, twice daily from D8.0 until D14.0 and once daily from D15.0 to D21.0, at which point all undiagnosed volunteers were treated. Matched samples for qRT-PCR were obtained at D7.5 only. For KCS, Oxford qPCR and TBS were performed at D6.5, twice daily from D7.0 until D14.0 and once daily from D15.0 to D21.0, at which point all undiagnosed volunteers were treated. Matched samples for qRT-PCR were obtained at D6.5, D7.0 and D7.5. TBS was performed as described [15].

### Sample preparation

EDTA-anticoagulated whole blood was collected on the indicated days following CHMI. For the Oxford qPCR assay, 2 mL of whole blood was filtered to remove leukocytes as described [17]. In KCS, samples were then frozen as 0.5 mL filtered blood volumes on-site (Nairobi, Kenya) and transferred to KEMRI-Wellcome Trust, Centre for Geographical Medical Research (Coast), Kilifi, Kenya for further processing. For VAC052, blood filtration, DNA extraction and qPCR were performed immediately on fresh samples onsite (Oxford, UK) to allow same-day monitoring. For the ‘standard volume’ qRT-PCR assay, 50 μL of whole blood was aliquoted into 2 mL lysis buffer (bioMérieux), which was then mixed and frozen onsite at −80°C as described [14]. For qRT-PCR from DBS cards (KCS study alone), 50 μL of EDTA-anticoagulated whole blood was pipetted onto a standard DBS card (Whatman), which was subsequently dried, desiccated and frozen onsite as described [14]. ‘Large volume’ qRT-PCR samples were prepared in duplicate by adding 0.5 mL whole blood to 20 mL lysis buffer (bioMérieux), after which the samples were frozen onsite and stored as for the standard samples. qRT-PCR samples were then transported on dry ice by courier to UW as lysis buffer-preserved, frozen samples (for standard and large volume aliquots) or as desiccated, frozen DBS cards.

### DNA extraction and qPCR (Oxford protocol)

Briefly, DNA was extracted from 0.5 mL of filtered blood, and 10% of the extracted DNA was used per qPCR reaction (in triplicate) as previously reported [9]. Based upon results obtained using a dilution series of microscopically counted cultured parasites, this method has a LLQ, (defined as the parasite density at which the assay %CV <20%) of 20 p/mL. Counted parasite dilution series results suggest that the LLD (i.e., a probability of >50% of ≥1 positive result among three replicate PCR reactions) is ~5 p/mL. Results were reported as ≥ LLQ when ≥20 p/mL and ≥ LLD when in the 5–19 p/mL range.

### Total nucleic acid extraction and qRT-PCR (UW protocol)

After storage at −80°C, standard volume liquid samples were extracted as described [14]. DBS samples were laser cut, incubated with 2 mL lysis buffer with continuous shaking overnight and then the lysis buffer was transferred to the m2000sp (Abbott Molecular, Inc). Large volume samples were completely thawed, and 1 mL of the 20.5 mL solution was transferred to the m2000sp instrument for extraction using the same parameters as for standard volume samples. Extraction and qRT-PCR for all specimens were performed as previously reported [14] with the RNA standard curve extended into the range corresponding to 1–20 p/mL. Standard volume and DBS samples were tested in singlet while large volume samples were tested in duplicate (2 × 0.5 mL = 1 mL sample). Results were reported for standard volume and DBS samples to 20 p/mL (≥LLQ) with ‘low positive below the LLQ’ reported in the 10–20 p/mL range (≥LLD). Large volume samples were reported to a quantitative limit of 2 p/mL (≥LLQ) with ‘low positive below the LLQ’ reported for samples in the 1–2 p/mL range (≥LLD).

### Statistics and modelling

Results were converted to log_10_ p/mL and evaluated using Microsoft Excel 2010 and GraphPad Prism 6. Where more than one replicate at a given time-point was available, the mean was used for analysis. For VAC052, ‘clinical sensitivity’ at D7.5 was calculated as ‘true positives/(true positives + false negatives)’ and ‘clinical specificity’ as ‘true negatives/(true negatives + false positive)’ using the study protocol-defined definitions of malaria infection as the gold standard (NAA *et al.,* in preparation). The VAC052 study protocol-defined definition of malaria infection included persons meeting any one of the three following scenarios: 1) no symptoms but with a positive TBS and at least one sample qPCR positive >500 p/mL; 2) malaria-related symptoms and a positive TBS; or, 3) malaria-related symptoms, a negative TBS and a least one positive qPCR result >500 p/mL. Vaccinees were defined as demonstrating a ‘significant delay to diagnosis’ if the time to diagnosis (TTD) was ≥2 × the standard deviation in days after the mean TTD for unimmunized control volunteers.

For KCS, ‘clinical sensitivity’ was calculated for the NAT results at D6.5. In addition, ‘cumulative clinical sensitivity’ was also calculated using results obtained until D7.0 (i.e., D6.5-7.0) and until D7.5 (i.e., D6.5-7.5). The KCS study protocol-defined definition of malaria infection was used as the gold standard [15] and included persons meeting any one of the three following scenarios: 1) no symptoms but positive TBS; 2) malaria-related symptoms and a positive TBS; or, 3) malaria-related symptoms and a negative TBS.

All results exceeding the LLD were used for all sensitivity and specificity comparisons. Data ≥ LLQ for each assay were assessed to determine correlation (using two-tailed Spearman test) and were plotted using Bland-Altman charts to determine quantitative bias.

Results were used to calculate LBI for individual volunteers using the peak first-cycle parasitaemia method (highest NAT value between D6.5-7.5 multiplied by the blood volume (volunteer weight × 70 mL blood/kg) [11]. A value of 1.0 was added to all LBI values to allow log-transformation of negative data points.

## Results

### Subject demographics

Samples were collected from 32 subjects in the VAC052 study and 28 subjects in the KCS study; demographics of volunteers are shown in Table 1. In the KCS study, whilst all volunteers were successfully infected, one volunteer demonstrated control of parasitaemia and remained undiagnosed until D21 [15]; data from this volunteer was excluded from sensitivity and specificity analyses.

**Table 1:** **Subject characteristics**

**Study**	**VAC052**	**KCS**
Number subjects	32	28
Mean age, years (range)	27 (19–45)	25 (19–31)
Sex	11 females, 21 males	11 females, 17 males
Site	Centre for Clinical Vaccinology & Tropical Medicine, Oxford, UK	KEMRI Centre for Clinical Research, Nairobi, Kenya

### Large volume assay accelerates early NAT diagnosis

Starting with samples from D7.5 in VAC052, Oxford qPCR (0.5 mL DNA) and UW-based standard (0.05 mL RNA/DNA) and large volume (0.5 mL RNA/DNA) qRT-PCR assays were performed to see if increasing the volume of blood tested by qRT-PCR enhanced sensitivity. This sample set included 32 persons, 27 of whom developed patent parasitaemia by D21, five of whom did not develop malaria infection by D21, and six of whom showed a significant delay to diagnosis (NAA *et al.*, in preparation). Of the 27 samples from subjects who became positive collected on D7.5, 19/27 were positive (≥LLD) by Oxford qPCR, 20/27 by UW standard volume qRT-PCR and 24/27 by UW large volume qRT-PCR. The only subjects negative by all assays were those who either did not develop malaria infection and three of six who showed a significant delay to diagnosis as noted above. Overall ‘clinical sensitivity’ (see definition in Methods) was 70.4% for the Oxford qPCR, 74.1% for the UW-based standard volume qRT-PCR and 88.9% for the large volume qRT-PCR assay; specificity for all assays was 100% (Table 2).

**Table 2:** **VAC052 sensitivity and specificity by assay for values ≥ LLD**

**Diagnosis by method (blood volume)**	**Sensitivity**	**Specificity**
UW large volume qRT-PCR (0.5 mL)	88.9%	100%
Oxford qPCR (0.5 mL)	70.4%	100%
UW standard qRT-PCR (0.05 mL)	74.1%	100%

**Figure 1:**
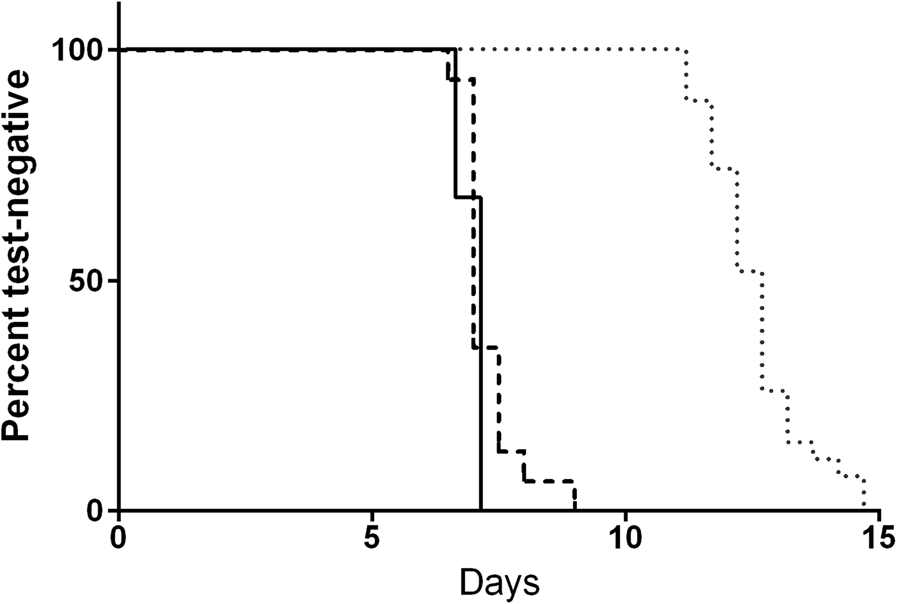
**Time to positivity for microscopy, Oxford qPCR and large volume qRT-PCR in KCS.** Kaplan-Meier survival curve representing the rate of conversion from negative to positive NAT results by test method (>=LLQ). Solid line, UW large volume qRT-PCR; heavy dashed line, Oxford qPCR; light dotted line, microscopy diagnosis.

Since the larger volume RT-PCR assay showed increased sensitivity at D7.5 in VAC052, the next step was to test blood from 27 subjects sampled during the first cycle of expected parasitaemia in the KCS trial (D6.5-7.5). All 27 subjects included in the analysis developed positive *P. falciparum* infections by TBS by the end of the trial [15]. Since 100% of the subjects were positive by the gold standard definition, specificity of the assays could not be compared. The large volume qRT-PCR assay was most sensitive in this dataset, followed by the Oxford qPCR, the standard volume qRT-PCR and the dried blood spot (DBS) qRT-PCR (Table 3 and Figure 1). On D6.5, the large volume qRT-PCR assay detected the highest number of positive specimens (10/27), followed by the Oxford qPCR (7/27) and the UW standard volume qRT-PCR assay (1/27). Amongst samples positive on D6.5, all large volume qRT-PCR (n = 10) and standard volume qRT-PCR assay samples (n = 1) were quantifiable within the limits of the assay (i.e., ≥LLQ), whereas all samples positive by Oxford qPCR (n = 7) were in the ‘low positive’ range (i.e., ≥LLD). By D7.0, most samples were qualitatively positive by all assays (27/27 by large volume qRT-PCR, 26/27 by Oxford qPCR, 19/27 by standard volume qRT-PCR and 14/26 by DBS qRT-PCR), which increased further by D7.5. A large proportion of samples tested by Oxford qPCR were identified as ‘low positives’ (i.e., LLQ > result ≥ LLD) (27/60 positive qPCRs on D6.5-D7.5) whereas this was less common amongst qRT-PCR positive samples (0/61 large volume qRT-PCR, 3/43 standard volume qRT-PCR, 5/35 DBS qRT-PCR). Finally, the large volume qRT-PCR and the Oxford qPCR assays became positive (≥LLD) on average on D7.0 compared to D12.5 by microscopy (Figure 1), a 5.5-day lead time over microscopy.

**Table 3:** **KCS sensitivity by assay for values ≥ LLD**

Diagnosis by method (blood volume)*	**D6.5**	**D7.0**	**D7.5**
UW large volume qRT-PCR (0.5 mL)	37.0%	100.0%	100.0%
Oxford qPCR (0.5 mL)	25.9%	96.3%	100.0%
UW standard qRT-PCR (0.05 mL)	3.7%	70.4%	88.9%
UW DBS qRT-PCR (0.05 mL)	0.0%	50.0%	84.6%
	n = 27 (n = 26 DBS)		

*KCS malaria diagnosis definition by D21 as the gold standard comparator.

### Comparison of parasite densities determined by different assays

**Figure 2:**
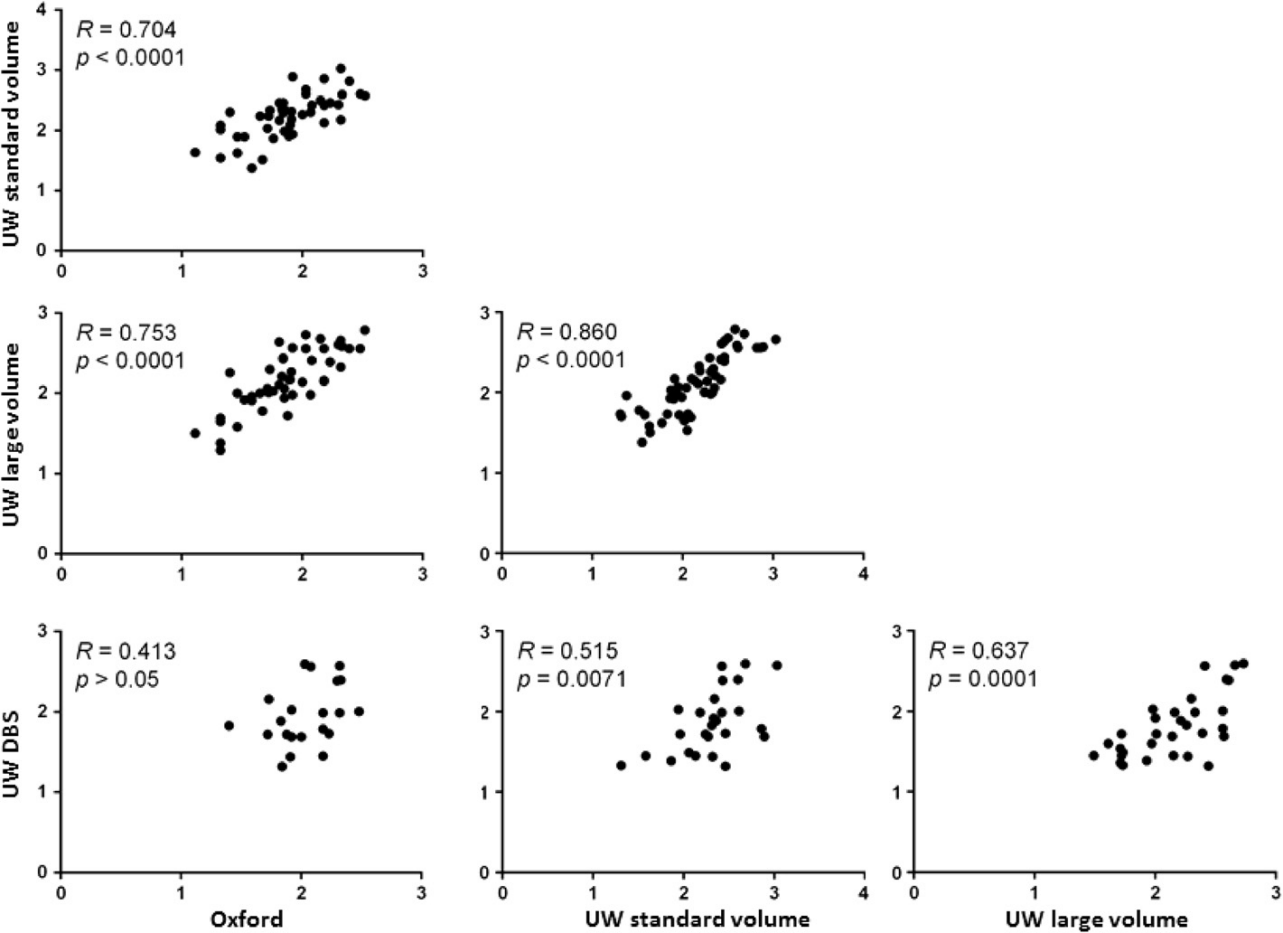
**Correlation analyses.** Data were compiled from all samples in both clinical trials where two methods produced results ≥ LLQ. Paired results were plotted as shown; Spearman rank correlation (R); two-tailed *p* value. Units for x and y axes are log_10_ p/mL whole blood as determined for paired samples by the methods listed on each axis.

**Figure 3:**
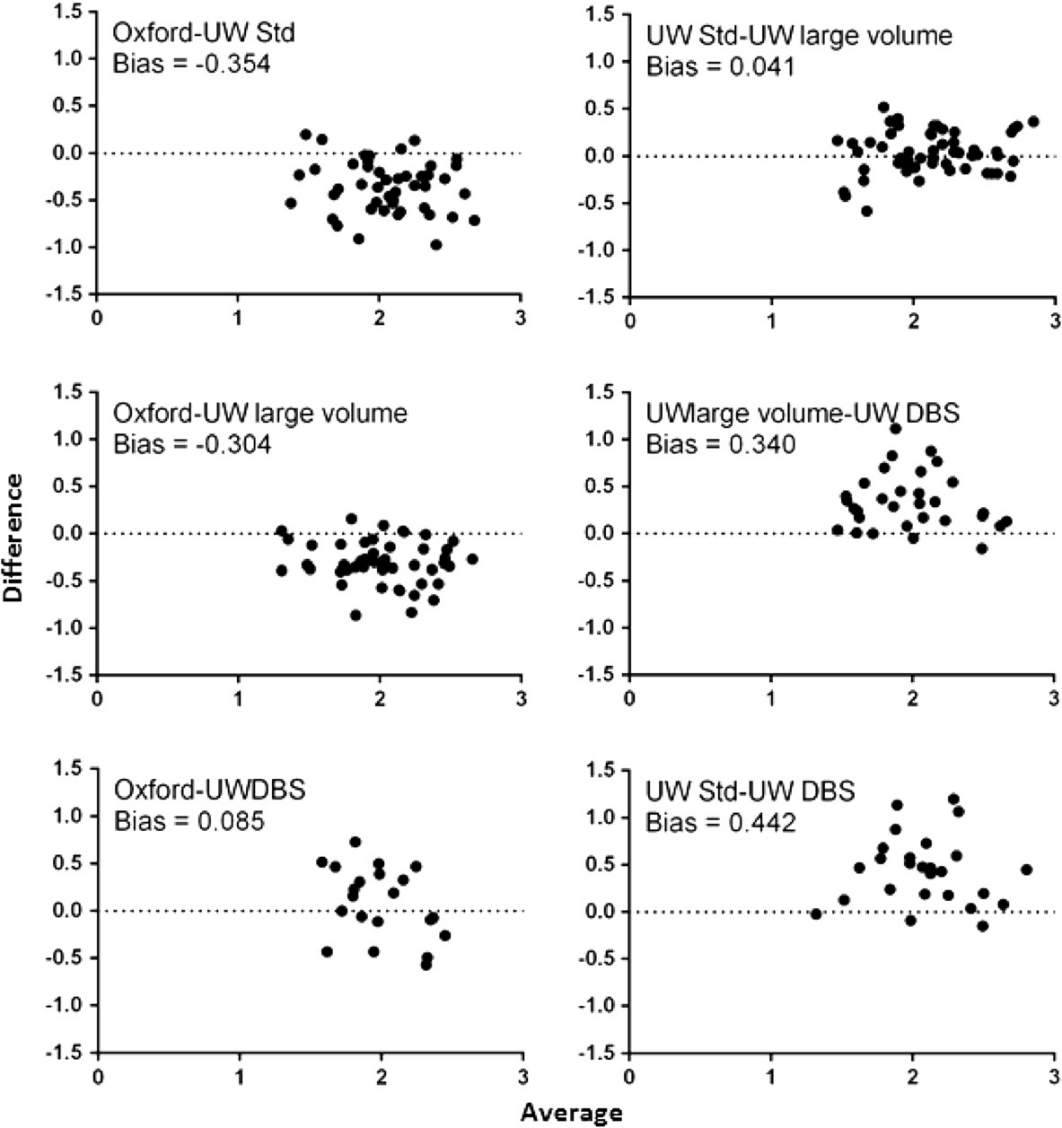
**Agreement analyses.** Data were compiled from all samples in both clinical trials where two methods produced results ≥ LLQ and plotted using a Bland-Altman (difference) chart showing the average result (x-axis) and the difference between results (y-axis) for each sample. The labels indicate the mathematical order used to calculate difference. Units for x- and y-axes are log_10_ p/mL whole blood as determined for paired samples by the methods listed on graph. The average difference from a value of 0.0 log_10_ p/mL equals the bias.

Next, an inter-assay comparison of quantitative results was performed. All tests that generated results ≥ LLQ were included. With the exception of the pairing of Oxford qPCR results with DBS qRT-PCR results, all datasets showed statistically significant concentration-dependent correlation with one another (Figure 2). The strongest correlation was between large and standard volume qRT-PCR assays. While Oxford and UW assays showed strong correlation, when comparisons were made using Bland-Altman plots to look for quantitative agreement and bias, a consistent quantitative shift between centers was observed (Figure 3), consistent with a previous report showing qualitative agreement of the Oxford data with that of other CHMI centers including UW, but a consistent quantitative shift at Oxford due to blood filtration and calibration differences [5]. DBS qRT-PCR results also diverged from UW liquid results, and this shift resulted in apparent alignment between Oxford qPCR and UW DBS qRT-PCR results.

### Effects of assay sensitivity on estimation of LBI and prediction of outcome

In VAC052, the purpose of the study was to assess the efficacy of novel vaccines targeting the pre-erythrocytic infection, a surrogate of which is LBI. A comparison was undertaken of LBI estimated from the maximum qPCR value obtained with Oxford qPCR between D6.5 and D7.5 (four data-points: D6.5, D7.1, D7.3, D7.5; the first cycle peak method [11]) and the UW large volume qRT-PCR result at D7.5 only. Sterilely-protected volunteers were NAT negative by all assays and were excluded from analysis. Comparison of the ability to calculate LBI using different datasets is shown in Table 4. Although LBI could be *estimated* for 81% of participants (n = 21) using Oxford qPCR data ≥ LLD, LBI could only be *quantified* using Oxford qPCR data ≥ LLQ for 67% of participants (n = 18). LBI could be *quantified* using UW qRT-PCR data ≥ LLQ in 85% of participants (n = 23).

**Table 4:** **Comparison of ability to estimate liver to blood inoculum (LBI) using peak method for participants in VAC052 and KCS: qRT-PCR** ***vs*** **Oxford qPCR methods**

**Study**	**LBI**		**Oxford qPCR: n (%)**			
			**Able to estimate using data ≥ LLQ**	**Able to estimate using data ≥ LLD**	**Not able to estimate using data ≥ LLQ**	**Not able to estimate using data ≥ LLD**
**VAC052** (n = 27)	**UW large volume qRT-PCR: n (%)**	Able to estimate using data ≥ LLQ	18 (67%)	22 (81%)	5 (19%)	1 (4%)
		Not able to estimate using data ≥ LLQ	0 (0%)	1 (4%)	4 (15%)	3 (11%)
**KCS** (n = 28)	**UW large volume qRT-PCR: n (%)**	Able to estimate using data ≥ LLQ	20 (71%)	28 (100%)	8 (29%)	0
		Not able to estimate using data ≥ LLQ	0	0	0	0

Sterilely protected VAC052 volunteers were NAT negative by all assays and were excluded from the analysis.

In the KCS study, LBI for the study publication was calculated with simple linear regression using Oxford qPCR values [15] but could not be calculated with simple linear regression using qRT-PCR data since samples were taken at only three first-cycle timepoints. For the purposes of comparison, LBI was therefore estimated here with the first cycle peak method using Oxford qPCR data ≥ LLQ and ≥ LLD and large volume RT-PCR data ≥ LLQ (Table 5). Use of qRT-PCR increased the proportion of KCS volunteers for whom LBI could be calculated more accurately using data > LLQ (Table 4).

**Table 5:** **Estimations of liver to blood inoculum (LBI) for KCS study**

**LBI**	Peak method*			
	**≥ LLQ**		**≥ LLD**	
	**Min**	**Max**	**Min**	**Max**
Oxford qPCR	97,608**	1,396,941**	33,768	1,396,941
UW large volume qRT-PCR	37,800	1,855,630	37,800	1,855,630

*Units are total number of parasites estimated to be released from the liver.

**n = 20 (8 volunteers had negative LBI). All other analyses n = 28.

## Discussion

In this study, an ultrasensitive *P. falciparum* 18S rRNA qRT-PCR was tested to determine if this approach could provide earlier and more quantitative data for CHMI modelling. The samples tested were from timepoints (D6.5-7.5) that coincide with the release of erythrocyte-stage parasites from the liver. During this early period, the absolute number of blood-stage parasites in a person following standard CHMI is extremely low (e.g., ~3×10^5^ erythrocyte-stage parasites emanating from ~14 infected hepatocytes based on [17-20] and likely lower with partial liver-stage efficacy of vaccine or drug treatments or with reduced sporozoite inoculum). Given the ~5 L blood volume of a 70 kg adult, the number of parasites estimated to be released by a single infected hepatocyte (~20,000) will result in a peak parasitaemia of ~4 p/mL in the first cycle. This level is not reliably detectable or quantifiable using previously described NATs. Moreover, parasite egress from the liver does not happen all at once and instead infected hepatocytes likely release merosomes more gradually starting on D5-6 [12,13]. As such, while assays capable of detecting 20 p/mL may detect all infected persons by the time that release from the liver is complete (e.g., D7.5), more sensitive assays may be better at defining the onset of release and the start of the erythrocytic stage.

The UW large volume qRT-PCR assay accelerated early malaria diagnosis compared to other assays. If the large volume qRT-PCR was applied at all pre-diagnosis timepoints post-CHMI, it is likely that the increased sensitivity could speed diagnosis if used in real time as a criterion to initiate treatment in CHMI studies assessing pre-erythrocytic drugs and vaccines. Data generated from such an approach could improve the accuracy of LBI estimation in those trials where early timepoints (up to D7.5) were frequently negative by standard assays [8,9]. In studies where early timepoints are qualitatively positive by Oxford qPCR or standard UW qRT-PCR, the large volume qRT-PCR could improve the accuracy of absolute LBI albeit at an increased reagent cost. However, given variability in the challenge inocula used in mosquito sporozoite CHMI studies, analyses are usually restricted to intra- not inter-study data, so the absolute qPCR or qRT-PCR (and calculated LBI) values are not as critical for comparisons between studies. If there is a move toward more CHMI trials using highly standardized inocula (e.g., injection of PfSPZ Challenge [3]), then it may be possible to perform inter-trial comparisons where use of the more sensitive large volume qRT-PCR method at multiple sites could be especially useful. Ultrasensitive diagnosis using this type of approach could also be useful in studies of Duffy-negative subjects challenged with large numbers of *Plasmodium vivax*-infected mosquitoes since these subjects do not develop patent parasitaemia but can have detectable parasite nucleic acids in their blood following successful liver-stage development and abortive erythrocyte infection (S. Herrera, pers. comm.). A deterrent to using the large volume qRT-PCR as performed here is the cost of the 20 mL of lysis buffer used to stabilize each 0.5 mL blood sample. Ongoing studies suggest 18S rRNA from 0.5-1.0 mL of blood can be protected from degradation by storage in 2 mL of lysis buffer provided that a sub-aliquot (e.g., 250 μL if 0.5 mL) is diluted in additional lysis buffer (1.75 mL) once thawed for extraction to maintain a standardized blood-to-lysis buffer ratio, although additional studies are required to confirm stability during storage and transport (SCM, pers. comm.). The above observations were made using quantitative data ≥ LLQ for each assay. When the LBI was calculated using all data (including data inferred on samples below the LLQ but ≥ LLD), there were minimal differences between the proportions of individuals with positive LBI calculated using the large volume qRT-PCR and the Oxford qPCR approaches. Thus, while preferable to use most accurate data (i.e., ≥LLQ), given the added cost of the large volume qRT-PCR sample stabilization, it may be suitable to use standard qPCR or qRT-PCR methods and include values < LLQ but ≥ LLD when calculating LBI endpoints.

With respect to quantitative agreement and correlation between assays, all methods generated data with a high degree of concentration-dependent correlation. The liquid-based assays at UW had the highest degree of quantitative agreement by Bland-Altman analyses, while the Oxford qPCR showed a consistent quantitative shift between Oxford qPCR and both liquid sample-based UW qRT-PCR assays. This quantitative shift was likely due to loss of parasites during Oxford filtering of whole blood and to differences in the matrix used for plasmid DNA calibrators at Oxford, as reported [5]. The UW DBS qRT-PCR also diverged from the UW liquid-based samples, likely due to incomplete recovery of the target from the DBS surface [14]. The apparent alignment between Oxford qPCR and UW DBS qRT-PCR results is likely due to losses in both assays but this alignment should not be used to consider the assays equivalent as both centers are working to reduce such losses to align these assays with the liquid qRT-PCR results (SCM, pers. comm.).

The factors that limit the ability of DNA-based qPCR *versus* total nucleic acid-based qRT-PCR to achieve increased sensitivity vary as follows. Since the Oxford qPCR detects relatively few 18S rDNA copies per parasite, this assay requires a PCR that can detect just a few copies of the 18S rDNA per reaction [9]. While single copy detection is achieved in some HIV-1 NATs [21,22], detection of any molecular target at one to 20 copies per reaction generally occurs beyond 40 cycles of PCR (or RT-PCR), which is nearing the performance limits for most assays. For the Oxford qPCR, the LLD is reached at ~41 PCR cycles. As such, in the Oxford qPCR, the relatively few target sequences present dictate the LLD. For the UW qRT-PCR [14], ~3,500 18S rRNA copies are detectable in each ring-stage parasite. Thus, at the published LLD for the UW qRT-PCR assay, when a single parasite is present in 0.05 mL of whole blood, there are 3,500 copies of the 18S rRNA target that are detected at ~33.5 cycles. Thus, in qRT-PCR, the LLD is dictated by whether or not a single intact parasite is present in the sampled blood volume – if a single parasite is present then the 18S rRNA load for even one parasite is readily detectable.

In this study, qRT-PCR was also performed on whole blood preserved on DBS cards. While the quantitative DBS data showed some target loss compared to liquid samples resulting in the lowest overall sensitivity between methods across all sampling days, sensitivity on D7.5 (84.6%) was comparable to standard qRT-PCR (88.9%) indicating that DBS-based qRT-PCR is suitable for pre-patent diagnosis well ahead of microscopic diagnosis. These samples were collected and initially stored in a field setting with the use of desiccant and humidity indicators and then were shipped to the UW testing facility, where they were processed by laser cutting and qRT-PCR. For studies where early (D6.5-7.5) diagnosis is not as critical, DBS qRT-PCR for malaria may offer an attractive option for field studies since the samples are conveniently collected, stored and shipped.

In summary, this report demonstrates that qPCR and qRT-PCR methods with analytical sensitivities of ~20 p/mL are sufficient for most CHMI purposes. At timepoints when directly calculated LBI is desired, an ultrasensitive large volume qRT-PCR may be most useful.

## Abbreviations

AMA1: Apical membrane antigen 1
CS: Circumsporozoite protein
CHMI: Controlled human malaria infection
C_T_: Cycle threshold
DBS: Dried blood spot
KEMRI: Kenyan Medical Research Institute
LBI: Liver-to-blood inoculum
LLD: Lower limit of detection
LLQ: Lower limit of quantification
MVA: Modified vaccinia virus Ankara
NASBA: Nucleic acid sequence-based amplification
NAT: Nucleic acid test
PCR: Polymerase chain reaction
qPCR: Quantitative PCR
qRT-PCR: Quantitative reverse transcription PCR
rRNA: Ribosomal RNA
rDNA: ribosomal RNA-coding genes
ME-TRAP: Thrombospondin related adhesion protein
TTD: Time to diagnosis
UW: University of Washington
